# The Impact of Parameter Identification Methods on Drug Therapy Control in an Intensive Care Unit

**DOI:** 10.2174/1874431100802010092

**Published:** 2008-05-27

**Authors:** Christopher E Hann, J. Geoffrey Chase, Michael F Ypma, Jos Elfring, NoorHafiz Mohd Nor, Piers Lawrence, Geoffrey M Shaw

**Affiliations:** 1 Centre of Bio-Engineering, Department of Mechanical Engineering, University of Canterbury, Christchurch, New Zealand; 2 Control Systems Technology Group, Department of Mechanical Engineering, Eindhoven University of Technology, New Zealand; 3 Department of Intensive Care, Christchurch Hospital, Christchurch, New Zealand

## Abstract

This paper investigates the impact of fast parameter identification methods, which do not require any forward simulations, on model-based glucose control, using retrospective data in the Christchurch Hospital Intensive Care Unit. The integral-based identification method has been previously clinically validated and extensively applied in a number of biomedical applications; and is a crucial element in the presented model-based therapeutics approach. Common non-linear regression and gradient descent approaches are too computationally intense and not suitable for the glucose control applications presented. The main focus in this paper is on better characterizing and understanding the importance of the integral in the formulation and the effect it has on model-based drug therapy control. As a comparison, a potentially more natural derivative formulation which has the same computation speed advantages is investigated, and is shown to go unstable with respect to modelling error which is always present clinically. The integral method remains robust.

## 1 INTRODUCTION

Therapy guidance using physiological models is a growing trend in bio-engineering [1-8]. In general, the idea is to use parameter identification to identify patient specific parameters then use these parameters to predict future dynamics, and in particular, individual patient response to therapy. For example, glucose control in the intensive care unit (ICU), has been dramatically improved by using a glucose-insulin model to optimize insulin doses and changes of nutrition [2, 9-14]. A glucose control protocol SPRINT (specialized reduced insulin nutrition table) has changed clinical practice in the Christchurch Intensive Care Unit [14]. The result is tight control of blood glucose with a 32% mortality reduction. Parameter identification is thus an important part of the overall process, as the identified parameters affect the overall therapy prediction. There are many methods for parameter identification, most of which are some variation of the standard non-linear regression [15]. These methods include gradient descent [16, 17], Bayesian with many starting points [18, 19] and hybrid approaches [20, 21].

The problem with these standard non-linear regression approaches is that they all typically require many forward solutions and starting points to ensure robustness. In a model-based therapeutics approach [2, 9-14] parameter identification can occur every 1-2 hours over periods of up to one or several weeks, for many patients. A Monte Carlo method, taking into account sensor error and error in fixed population parameters to optimize therapy selection, also significantly increases the number of parameter identifications required each time. Similar Monte Carlo approaches to optimizing such protocols in a virtual patient simulated trial [10, 14, 22] are also computationally intense for the same reasons.

An integral-based parameter identification method has been developed [23] and extended to other physiological systems [24-27], that avoids the need for any forward simulations. It can thus dramatically reduce the computation required. These integral methods are therefore well suited to model-based control applications requiring real-time parameter identification. For example, agitation sedation control [28, 29], fluid therapy and inotropic drug administration for improved cardiac management [25] and control of neuromuscular blockade in general anaesthesia [21, 30]. This paper investigates different computationally fast formulations that don’t require forward simulations and in particular examines the impact of these methods on physiological model-based glucose control.

These issues are illustrated and tested with respect to noise and modelling error using an exemplar glucose-insulin model that has been extensively validated over many clinical trials [2, 9-14, 22, 23, 31-40]. The glucose-insulin model and methods are tested using retrospective clinical data. Several practical issues that arise in clinical implementation are addressed, to highlight issues of performance and stability.

Finally, a new model-based control method for metabolic control is presented, that combines a non-invasive continuous glucose sensor (CGMS) [41] with current standard glucometer sensors [42]. This method is shown to provide a potentially significant improvement in glucose control in simulation that warrants further clinical investigation in the future.

## 2 METHODOLOGY

### 2.1 Glucose-Insulin Model

The glucose-insulin model is defined [11-13, 23]:

(1) G.=−pGG−SItGQ1+αGQ+P

(2) $$\dot{Q}=-k\left(Q-1\right)$$

(3) $$\dot{I}=-\mathrm{nI}+\frac{u}{V}$$

(4) P=Pt+pGGE

where *G(t)* is the plasma glucose concentration (mmol/L); *G_E_* the equilibrium level of plasma glucose concentration (mmol/L); *Q(t)* the interstitial insulin; *I(t) *the concentration of the plasma insulin above basal level (mU/L); *P(t) *the exogenous glucose infusion rate (mmol/(L min)); *u(t) *the insulin infusion rate (mU/min); *V* the assumed insulin distribution volume (L); *n * the delay in interstitial transfer of insulin (min^-1^); *p_G_* the fractional clearance of plasma glucose at basal insulin (min^-1^); *S_I_* the time-varying insulin sensitivity (L/mU min); *k* the parameter controlling the effective half life of insulin (min^-1^); and α*_G_* the Michaelis-Menten parameter for glucose clearance saturation. For more details on the construction and physiological interpretation of the model Equations (1)-(3) see [11-13, 23].

### 2.2 Parameter Identification

#### 2.2.1 Integral Method

For the glucose-insulin Equations (1)-(3), a similar integral-based parameter identification method to [23] is applied. The parameters *α_G_*, *k*, *n* and* p_G_* in Equations (1)-(3) are held constant at the population values based on prior studies and sensitivity analysis [23]:

(5) $$\alpha_{G}=\frac{1}{65},k=0.0099,n=0.16,p_{G}=0.01$$

Similarly, the parameter *G_E_* is held at the mean glucose of each patient. The nutritional carbohydrate input appearance rate, *P(t)* in Equations (1) and (4) is also held constant, but may change with respect to time for different patients. The exogenous insulin *u(t)* is defined:

(6) $$\begin{array}{c} u\left(t\right)=u_{I}+u_{B},0\leq t\leq 1\\ =u_{I},1\leq t\leq 60 \end{array}$$

where *u_I_* (mU/min) is the constant infusion rate over 1 hour and *u_B_* (mU/min) is the amount of bolus given over one minute. The parameter *S_I_* is insulin sensitivity and is assumed unknown. Integrating Equation (1) from 0 to *t* yields:

(7) Gt−G0=−pG∫0tGdt−SI∫0tGtQtdt+∫0tPdt

(8) Q=Q1+αGQ

Choosing *n* values of time, $t=t_{1},...,t_{n},\in \left[0,60\right],$ where   $0<t_{1}<\cdot \cdot \cdot <t_{n},$ a set of *n* equations are formulated:

(9) Gti−G0=−pG∫0tiGdt−SI∫0tiQtGtdt+∫0tiPdt,i=1,...n

where   $\bar{Q}\left(t\right)$ is defined in Equation (8). To avoid any error in G(0) potentially propagating through the equations, *G_0_* = *G*(0) is assumed unknown and is identified along with *S_I_*. Equations (9) can be written as a matrix system:

(10) ∫0t1Q¯Gdt⋅⋅⋅∫0tnQ¯Gdt−1⋅⋅⋅−1SIG0=−Gt1−pG∫0t1Gdt+∫0t1Pdt⋅⋅⋅−Gtn−pG∫0tnGdt+∫0tnPdt

where *G *is a continuous approximation to the measured glucose [23] and the integrals are evaluated by the trapezium rule. Equation (10) can be solved by linear least squares to determine *S_I_* as a constant over any period. Thus, *S_I_* may be identified as piecewise constant.

For glucose control in the Intensive Care Unit (ICU), Equation (1) is utilized over periods of 1 hour [11, 13] and glucose is measured on the hour. For two glucose measurements *G*_0_ = *G*(0) and *G*_60_ = *G*(60), the function *G*(*t*) in Equation (10) can be approximated by a straight line [23]. For a given infusion *u_I_* or bolus *u_B_* in Equation (6), nutritional input *P*(*t*) and glucose measurements *G_0_* and *G_60_*, the solution to Equation  (10) determines the required insulin sensitivity. However, note that a similar approach could be used if glucose is measured more frequently.

#### 2.2.2 Similar Approach with the Derivative

A similar, potentially simpler, approach to the parameter identification of Equations (7)-(10) is to use the original differential Equations (1)-(3), rather than an integral formulation. For a given set of values, $t=t_{0},...,t_{n},n+1$ equations can be formulated:

(11) G⋅ti=−pGGti−SIQtiGti+P,i=0,...,n

where *t_0_*=0. The analogous matrix system to Equation (10) is defined:

(12) Qt0Gt0⋅⋅⋅QtnGtnSI=P−pGGt0−G.t0⋅⋅⋅P−pGGtn−G⋅tn

where $\dot{G}\left(t_{i}\right)$ are determined by standard finite differences. Equation (12) can be solved by linear least squares to determine *S_I_*.

This method applies gradients which is similar in concept to typical gradient descent methods. The major difference is that no forward simulations are required so like the integral method [23] it is a computationally fast way of identifying large numbers of *S_I_* or other time-varying parameters.

### 2.3 Controlling Drug Delivery

For the control of blood glucose *G*(*t*) in Equation (1), measurements are assumed to be taken every hour with a normally distributed absolute error of 7%, which is typical for a commercial glucometer [42]. Model-based control of glucose typically starts by taking two measurements *G_0_* and *G_60 _*at the times 0 and 60 minutes and computing the insulin sensitivity *S_I_* from Equation (10). The goal is to determine the required insulin infusion *u_I_* or bolus *u_B_* in Equations (1) and (6) that will bring glucose down to a target value *G_target_* in the next hour.

Let *S_I,_*_1_ be the solution of Equation (10) that determines the insulin sensitivity in the first hour. Define *S_I,_*_2_ as the insulin sensitivity in the second hour. In the ICU a patient’s condition can change rapidly as a result of a disease state or drug therapy. Therefore, *S_I_* can change significantly over time [2, 23]. Given *S_I,_*_1_ in the first hour, it is thus possible that *S_I,_*_2_ in the second hour may have changed. An approximation to the insulin sensitivity *S_I,_*_2_ in the second hour for predicting potential outcomes of an intervention at the end of hour 1, is defined ${\bar{S}}_{I,2}=S_{I,1}$ . As long as the true $S_{I,2}$ doesn’t change significantly from *S_I,_*_1_, this value ${\bar{S}}_{I,2}$ can be used to determine the insulin control input *u*(*t*) in Equation (1) that will bring the glucose to the target value of *G_target_*. Any significant changes will induce increasingly, unavoidable errors in the prediction.

First assume that *u_B_* = 0 in Equation (6) and that only constant insulin infusion in the second hour is used. An example is given in Fig. (**1**), which includes a “true” glucose response to an infusion of *u_I_* = 2 units over 1 hour with a nutritional input of *P*(*t*) = 0.03mmol/L/min. Insulin sensitivity *S_I,_*_1_ = 0.0008 (L/mU min), *S_I,_*_2_ = 0.001 (L/mU min), *G_E_*=4.5 mmol and the rest of the parameters in Equation (1) are given in Equation (5). The “measurement” points *G_0_*=8 and *G_60_*=7.26 are denoted by crosses (+) in Fig. (**1**) and the target glucose *G_target_*=5 mmol/L is denoted by a circle (o). No noise is added.

For simplicity, it is assumed that *S_I_* is precisely known in the first hour. In practice, either the solution to Equation (10) or Equation (12) would approximate * S_I_* . Assuming that ${\bar{S}}_{I,2}=0.0008$ in the second hour, the goal is to find the infusion  *u_I_* such that the numerical solution *G*(*t*) to Equation (1) with *S_I_=S_I,2_*, and initial conditions, {*G*(0)=*G_60_*, *Q*(0)=*Q_60_*, *I*(0)=*I_60_*} satisfies *G*(60)=*G_target_*. The values of *Q_60 _*and *I_60_* are determined by the evaluating the numerical solution to Equations (2)-(3) at  *t* = 60. Note that without loss of generality, the time at the beginning of drug intervention is assumed to be at 0 minutes and the target value is assumed to be at 60 minutes.

To determine *u_I_*, Equation (1) is solved numerically for a range of infusion values *u_I_*, and the resulting end glucose value is compared to the target value. The end glucose value is represented as a function ${\bar{G}}_{\mathrm{target}}\left(u_{I}\right)$ , and is defined:

(13) GtargetuI=G60,G≡solution of Equations 1−3 with ut=uI

The correct *u_I_* is denoted *u_I,target_* and is defined:

(14) uI,target≡uI:GtargetuI=Gtarget

where ${\bar{G}}_{\mathrm{target}}\left(u_{I}\right)$ is defined in Equation (13). Define the points:

(15) uI,i=i−1,i=1,...,7 Gtarget,i=GtargetuI,i,i=1,...,7

where *u_I_* is treated as a variable on the y axis. Fig. (**2**) shows the points $\left\{\left({\bar{G}}_{\mathrm{target},1},u_{I,1}\right),...,\left({\bar{G}}_{\mathrm{target},7},u_{I,7}\right)\right\}$ plotted as crosses (+). A cubic spline is then fitted to the data, which is shown as the solid line in Fig. (**2**). Evaluating the cubic spline at *G_target_* in Fig. (**2**) allows a good approximation to *u_I,target_* of Equation (14) with only 4 numerical solutions of Equations (1)-(3) required. This overall method for determining *u_I,target_* of Equation (14) is summarized in Fig. (**3**).

For this example, the target infusion was calculated to be *u_I_* = *u_I,target_* = 3.27*U*. The resulting glucose response with *S_I_* held constant at the approximate value of 0.0008 L/mU min is denoted by a dashed line in Fig. (**1**). This approximate glucose response hits the target *G_target_* as required. However, the “true” glucose response, which comes from using the output infusion *u_I,target_* in Fig. (**2**) with the true value of *S_I,2 _*= 0.001, slightly undershoots *G_target_* in Fig. (**1**). The end result in this case is still accurate, with an error of 8%. In practice both noise, modelling error and natural variation in *S_I_* can effect the accuracy of hitting the target glucose and is investigated in detail in the results.

### 2.4 Forward Simulation Based Methods and Summary

The most common approach to parameter identification as discussed in the introduction are methods that rely on many forward simulations. A standard non-linear regression least squares (NRLS) gradient descent algorithm was tested rigorously in [23]. Assuming a reasonable starting guess, the NRLS method was thousands of times slower than the integral method of [23]. Furthermore, local minima’s were often found so that the best insulin sensitivity estimate *S_I_* was not always found.

The problem of local minima’s in NRLS can always be corrected by starting at many starting points, like the method of Cobelli [19]. However, this dramatically increases the number of forward simulations. For example in [23], the integral method was 1000 times fast than the NRLS algorithm which started from one initial guess. If 10-100 starting points were used for the NRLS algorithm, which is quite typical to ensure accuracy (e.g. Cobelli [19]), the integral method would be 10000-100000 times faster than NRLS. The speed gain increases even further as the complexity of the model and number of fitted parameters increase, for example a cardiovascular model (e.g. [24, 26]).

For a model-based therapeutics approach [2, 9-14], the large number of forward simulations required in the NRLS approach is extremely costly, and is not feasible to implement. Note that an NRLS approach could be applied in the model-based control examples of this paper, and would give similar results to the integral method, but it comes at a considerable computational cost. Therefore, since the model-based therapeutics approach requires minimal computation, this paper focuses entirely on methods that do not require a forward simulation. The two methods considered are the derivative method of Equations (11) and (12) and the integral method of Equations (7)-(10).

The derivative approach is a commonly used concept, for example in gradient descent algorithms, and would therefore most likely be the more easily understood and derived method. It is also perhaps, a more natural way of proceeding, since the original differential equation model is written in terms of derivatives. Therefore, the derivative approach at first sight would appear to be the simplest to implement and potentially a reasonable way of avoiding forward simulations in the parameter identification part of the model-based control algorithm of Fig. (**3**).

However, as is shown in the results, the integral formulation, which in general is perhaps a less known and accepted way of representing a differential equation model; is in fact fundamental for reliable results. This phenomenon was also investigated in a related approach to parameter identification of a minimal cardiac model on clinical pulmonary embolism animal data [26], where even with perfectly smooth, model generated signals, a derivative approach went unstable. The integral approach on the other hand remained stable.

Hence, the main aim of this paper, is to investigate the effect of the two fast parameter identification integral and derivative based methods on the glucose-insulin model; and to better explain the importance of the integral in the formulation. Most importantly, this study is done in the context of model-based therapeutics and glucose control in the Christchurch Hospital Intensive Care Unit.

## 3 RESULTS AND DISCUSSION

This section reviews the implementation of the integral method for long term model-based glucose control and compares the method with the similar approach that is based on the derivative. It thus contrasts the difference in using integrals and derivatives for this type of bio-engineering inspired parameter identification. The robustness of each formulation is investigated with respect to measurement noise and modelling error, intervention period, and number of measurements used.

### 3.1 Glucose control in the Christchurch ICU

The glucose control protocol SPRINT [9, 10, 14, 32] is now used extensively in the Christchurch ICU. One of the keys to the success of SPRINT is the significant testing of model-based glucose control algorithms on “virtual” patients prior to implementation. The major physiological variable that is used to represent a “virtual” patient profile is the time varying insulin sensitivity *S_I_* = *S_I_* (*t*) profile in Equation (1) that can be identified from retrospective data.

The integral-based parameter identification method [23] allowed fast and accurate insulin sensitivity profiles to be constructed for long term patient data. These profiles allowed an accurate physiological representation of a patient’s metabolic dynamics over periods of up to 1-2 weeks [23], and were a fundamental element in the development of SPRINT [9, 10, 14, 32].

The insulin sensitivity profiles provide a means to simulate physiologically realistic time varying glucose response to different insulin and nutrition regimes. This approach thus provides a repeatable cohort for easy comparison of various protocols. It also gives insight into long term clinical performance, and, importantly, lets algorithms and methods be tested safely before clinical implementation.

Fig. (**4**) shows a comparison of the “virtual clinical trials” versus the clinical data from the SPRINT trial in the Christchurch ICU for the first 16,000 clinical measurements and 24,000 hours of control. The distributions for the “virtual trials” are very close to both the raw SPRINT data and a lognormal fit of the data. The results of the virtual patient trials of other protocols [43-45] (not shown) also match their reports. The tightness of the SPRINT results and good correlation of other protocols serves as a significant validation of the methods and approach.

To further illustrate the impact of SPRINT, Fig. (**5**) shows a patient on SPRINT compared to a patient on a previously implemented clinical sliding scale in the Christchurch ICU. The measurements in both Fig. (**5a**) and (**5b**) are taken every hour, but the SPRINT patient is significantly better controlled than the patient on the original standard sliding scale. Note that the SPRINT patient also has 2-4 potentially contaminated measurements but still provides better control to a 4-6.1 mmol/L or similar target band than the retrospective data patient who is less acutely ill by APACHE II score.

### 3.2 Parameter Identification – Integral Versus Derivative

For ease of reference in this section and the following sections, Equations (7)-(10) are referred to as the Integral Method and Equations (11)-(12) are referred to as the Derivative Method. The methods are derived from the same set of differential Equations (1)-(3). Therefore, if no noise is present, it may be reasonable to suggest that they should perform equally well when identifying *S_I_*. In addition, neither requires the forward simulation used in most typical identification approaches. To test this assumption, the following set of parameters is considered:

(17) $$\begin{array}{c} P=0.08 mmol/\left(\text{Lmin}\right),U_{i}=\frac{100}{3}\text{mU}/\text{min},\\ U_{b}=0,S_{I}=0.0001L/\text{mU min} \end{array}$$

“Measured” glucose values at *t* = 0 and *t* = 60 are generated by numerically solving Equations (1)-(3) for various values of *G_E_*, and initial conditions *Q*_0_ with a fixed initial glucose of *G*_0_ = 5 mmol/L. Two main parameters sets are considered:

(18) $$G_{E}=4.5\text{mmol/L},0\leq Q_{0}\leq 20 mU/L$$

(19) $$Q_{0}=8\text{mU/L},3\leq G_{E}\leq 15\text{mmol/L}$$

In Equation (18), *Q*_0_ is varied in steps of 1 mU/L and in Equation (19), *G_E_* is varied in steps of 1 mmol/L. Figs. (**6**) and (**7**) show the results of the identified *S_I_* using the integral and derivative methods for each parameter set of Equations (18) and (19).

Fig. (**6**) shows that for *Q*_0_ < 5 mU/L the derivative method gives an *S_I_* value that is significantly different from the true value. In fact it becomes non-physiological and negative for *Q*_0_ = 0 and 1. The integral method, in contrast, remains stable. Fig. (**7**) shows a similar result with the derivative method rapidly diverging after *G_E_* = 4 mmol/L, and the integral method staying virtually constant.

The scenarios of Figs. (**6**) and (**7**) can be realized in practice whenever the insulin is cut off, so that *Q(t)* reaches low levels, followed by an increase of carbohydrate input (see example to follow). In particular, a negative *S_I_* would occur whenever the true *S_I_* is sufficiently low, so that the typical undershooting that occurs with the derivative method goes less than zero, as the example in Fig. (**6**) demonstrates.

#### 3.2.1 Model-based Glucose Control Example – Minimizing Insulin Infusion

To demonstrate the results of Figs. (**6**) and (**7**) in a clinical setting, a patient from the retrospective cohort of [23] is considered. The patient used is Patient 554, who was a female aged 20; type 1 diabetic; medical subgroup – Other Medical; APACHE II score - 26. Seven hours of data is analyzed, and Fig. (**8**) shows the time-varying insulin sensitivity for this period taken from [23]. Patient 554 also has the parameters:

(20) $$G_{E}=\text{4.5 mmol/}{\text{L and G}}_{0}=5.4 mmol/L$$

and all the other parameters are defined in Equation (5).

To begin the model-based control algorithm of Fig. (**3**), two glucose values are required in the first hour. These values are generated by solving Equations (1)-(4) with the parameters of Equation (5) and (20); an insulin infusion input of *u_I_* = 0.5 U , *u_B_* = 0 for *u*(*t*) in Equation (6); and initial conditions for insulin defined, by *I_0_* = 1 mU/min, *Q*_0_ = 1 mU/min. The target glucose in Step 4 of Fig. (**3**) is *G_target_* = 5mmol/L. Additional constraints for this example, are that the use of exogenous insulin *u_I_* is minimized and is only in steps of 0.5 U, and that when possible, the carbohydrate input *P*(*t*) , is the primary controller with a resolution of 0.01 mmol/L/min. Finally, it is assumed that for hour 6 the feed is increased to 0.06 mmol/L/min.

The identified insulin sensitivity for each of the derivative and integral methods is shown in Fig. (**9**), along with the true insulin sensitivity of Fig. (**8**). Notice that even without noise, both parameter identification methods deteriorate at hours 5 and 6, but the integral method is the most accurate. The absolute percentage errors of the methods for hours 1-6 in Fig. (**9**) are:

(21) $$\begin{array}{c} {\mathrm{error}}_{\text{derivative}}=\left[1.3,10.5,5.6,0.4,34,55.2\right]\left(\%\right)\\ {\mathrm{error}}_{\text{integral}}=\left[3.5,6.9,3.0,0.8,19.7,13.0\right]\left(\%\right) \end{array}$$

This deterioration is a result of low insulin levels which progressively removes the effect of *S_I_* on the glucose response, and thus the large errors in *S_I_* have a negligible effect on glucose control, which is shown in hours 1-6 of Fig. (**10**). This state of no insulin and very little carbohydrate input, of course could not be sustained for any significant period of time, as the patient would face malnutrition/starvation. Thus, the feed is increased at hour 6.

There is no reliable insulin sensitivity value from the prior hour due to the very low insulin levels. Therefore, a conservative infusion of 0.5 mmol/L/min is applied at hour 6 to identify insulin sensitivity so that the algorithm of Fig. (**3**) can be applied accurately in the following hour. Fig. (**9**) and Equation (21) show that the integral method identifies the insulin sensitivity quite accurately at hour 6 with an error of 13.0%, where the derivative method has a much larger error of 55.2%.

However, the significant under prediction of insulin sensitivity for the derivative method dramatically affects control. Fig. (**10**) shows that control in hour 7 for the derivative method is poor, with a 5.5 mU bolus predicted and an undesirable, and potentially dangerous, hypoglycaemia event of 3.61 mmol/L. This result corresponds to an error of 27.8% in the target glucose. On the other hand, the control based on the integral method is good with a final glucose value of 4.83 mmol/L, which corresponds to a 3.4% error. The results of Figs. (**9**) and (**10**) further confirm the observations of Figs. (**6**) and (**7**).

Note that the scenario of Fig. (**10**) is not uncommon in critical care and for the extended retrospective data set given in Shaw *et al. *[48], can occur several times daily. Therefore, the integral method allows more flexibility in the control protocol by not requiring insulin infusion to be on constantly, and is robust to sudden increases in the carbohydrate input.

#### 3.2.2 Model-Based Glucose Control – Constant S_I_ Approximation 

To further test the methods for a longer period and to demonstrate the practical, clinical issues associated with model-based control, another patient from the retrospective data of [23] is used. The patient is Patient 519, who was a male aged 69; type 2 diabetic; medical subgroup - General Surgical; APACHE II score - 29. The integral and derivative methods are compared based on a constant *S_I_*, which is taken to be the mean *S_I_* of patient 519. Similarly, the parameters *P*(*t*) and *G_E_* in Equations (1)-(4) are set constant at the mean nutritional input and mean glucose respectively of patient 519. The numerical values of the parameters are thus defined:

(22) $$S_{I}={9.28\times 10}^{-4}L/\text{mU min},P\left(t\right)=0.049 mmol/\left(\mathrm{Lmin}\right),G_{E}=5.84\text{mmol}/L$$

The rest of the model parameters are defined in Equation (5). Data for the first 3 days of patient 519 is used to test the predictive model-based glucose control of Fig. (**3**). Note that the protocol of minimizing the insulin, which was implemented in Fig. (**9**), is not used.  

To begin the model-based control algorithm of Fig. (**3**), two glucose values are required in the first hour. These values are generated by solving Equations (1)-(4) with the parameters of Equation (22); an insulin infusion input of *u_I_* = 1U , *u_B_* = 0 for *u*(*t)* in Equation (6); and initial conditions of *G*_0 _= 11.5 mmol/L, *I_0_* = 0 mU/min, *Q*_0_ = 0 mU/min. The target glucose in Step 4 of Fig. (**3**) is defined:

(23) $$G_{\mathrm{target}}=\text{max}\{G_{0}-1,5\}$$

where for each consecutive hour the initial conditions *G*_0_, *I*_0_ and *Q*_0_ are taken as the previously calculated *G*_60_, *Q_60_* and *I*_60_, as detailed in Step 2 of Fig. (**3**). Equation (23) ensures the reductions in glucose are not too large which clinically, may be undesirable for the patient.

Every hour that the algorithm of Fig. (**3**) is applied, a new infusion *u_I,target_* is defined for the next hour, which in turn defines a new glucose response, and so on as long as required. In this example, the final time is at 3 days or 72 hours, which gives 71 intervention periods since the first period is just a fitting period. Importantly, the size of the infusion cannot be greater than 6 Units [11, 13] for patient safety. To be physiologically realistic it must be also greater than 0. Therefore, the infusion *U_I, target_* in Fig. (**3**) is constrained:

(24) $$0\leq U_{I\mathrm{,target}}\leq 6\text{U}$$

The results of the algorithm of Fig. (**3**) for the integral method of Equations (7)-(10) are shown in Fig. (**11a**), where 7% uniformly distributed noise is placed on the hourly glucose measurements to mimic the sensor error in the glucometer [11, 13, 23]. All measurements in Fig. (**11a**) lie in the 4-6.1 mmol/L band showing that very tight glucose control is achieved when *S_I_* is constant. Fig. (**11b**) shows the results of using the derivative method of Equations (11)-(12) in place of the integral method in Step 3 of Fig. (**3**). Again all measurements lie in the 4-6.1 mmol/L band, showing there is virtually no difference between the methods.

The result of Figs. (**11a,b**) shows that for Patient 519, the parameter regimes of Equations (18) and (19) that caused instability for the derivative method in Figs. (**6**) and (**7**), were not realized. The mean value of *Q*(*t*) during this “virtual trial” of patient 519 was 16.5 mU/min. Examining Fig. (**6**), it can be seen that for these relatively high *Q*(*t*) values the derivative and integral methods behave similarly.

### 3.3 Model-Based Glucose Control – Time Varying *S_I_*

The results of Fig. (**11**) show that with continual insulin infusions over time the derivative and integral methods perform similarly in glucose control with hourly measurements of glucose. Therefore, since the main differences in control have already been investigated in Fig. (**10**), the comparison of the derivative and integral methods is discontinued in this section.

The insulin sensitivity profile of Patient 519 as fitted in [23] is highly dynamic, and the first three days are shown in Fig. (**12**). To demonstrate the practical aspects of model-based glucose control, the algorithm of Fig. (**3**) is applied to the time varying *S_I_* of Fig. (**12**) using the integral method. Note that in [38, 39] and Lin 2007 [49], the integral method has been well validated and proven for extensive numbers of virtual patients, therefore no further patients other than Patient 519 are tested in this paper.

The nutritional input *P*(*t*) is again held constant with all other parameters the same as given in Equations (5) and (22).

Fig. (**13**) gives the result for the integral method, which shows that glucose control is significantly worse than Fig. (**11**). The mean glucose and standard deviation of 5.58 ± 1.03 mmol/L with 67.57% of glucose values lying in the 4.0 to 6.1 mmol/L band. Very similar results are obtained for the derivative method, so these results are not shown.

The reason for this decrease in performance is explained by the insulin infusion graph of Fig. (**14**). There are significant periods in Fig. (**14**) where the insulin has reached the maximum of 6 Units/hour so effectively no added, but necessary, control is being applied in these periods and insulin effect is saturated [2, 36]. The solution to this problem has been to vary the nutrition, as well as the insulin [9, 10]. A fully developed and validated method for modulating both the nutrition and insulin in a model-based glycemic control system is detailed in [13, 14].

To demonstrate the essential concept the nutrition is dropped to 40% of the original value, whenever the insulin hits the upper limit of 6 units. A new insulin infusion is then calculated in Step 4 of Fig. (**3**) for this reduced nutrition. This simple rule results in a significant improvement in glucose control as shown in Fig. (**15**). The mean glucose is 5.32 ± 0.67 mmol/L with 76.14% of values lying between 4 and 6.1 mmol/L.

### 3.4 Combining CGMS with Glucocard Measurements

To demonstrate a new clinical application of the methods presented and to further investigate the comparison of the integral versus derivative approaches, a CGMS sensor is included in the model-based glucose control algorithm. The CGMS sensor measures glucose every 5 minutes with a measurement error that can be approximated by the formula [34]:

(25) $$G_{\mathrm{noise}}=\left(1+0.18\delta \right)G_{\mathrm{true}},\delta \equiv \text{ normal distribution}\left(\mu =0,\sigma =1\right)$$

Equation (25) gives a mean absolute error of 14%, which is typical for CGMS sensors [41, 50].

Blood glucose is still assumed to be measured hourly with a glucocard and 7% uniformly distributed noise in addition to the CGMS for comparison. To account for the extra noise in the CGMS and to give the greatest chance for an averaging effect on the errors, insulin sensitivity *S_I_* is fitted over the prior 1½ hours rather than 1 hour, as was presented in Fig. (**3**). The intervention period is also shortened to ½ hour to take advantage of the extra measurements from CGMS. The 1½ hour periods ensure that 2 glucocard measurements will always be available to fit *S_I_* when stepping along each interval of ½ hour.

The same algorithm of Fig. (**3**) is applied, except the integral and derivative methods are implemented over the longer 1½ hour period and the infusion *U_I,target_* in Step 4 of Fig. (**3**) is updated every ½ hour. A further change that is made is that 7% low frequency modelling error is added to the glucose measurements, as well as the normally distributed error in Equation (25). The final expression for noise is thus defined:

(26) $$G_{\mathrm{noise}}=\left(1+0.18\delta \right)\left(1-0.07\text{cos}\left(\frac{2\pi }{82}t\right)\right)G_{\mathrm{true}}$$

Equation  (26) reflects the fact that a higher resolution in measurements, trades off with both a higher amount of sensor error and importantly, modelling error.

The modelling error is caused by potentially missed dynamics in the glucose-insulin model of Equations (1)-(3). Simple oscillations are used as an initial proof of concept since low frequency oscillations have been often observed in both glucose and S_*I*_ [23]. However, further work must be done on real CGMS data to fully characterize the tradeoff’s in the error.

Fig. (**16**) shows the resulting glucose control for Patient 519 using the same parameters as used for Fig. (**15**). A significant improvement can be seen with a mean glucose of 5.03 ± 0.42 mmol/L and 98.55% of glucose values lying between 4 and 6.1 mmol/L.

Fig. (**17**) shows the first 12 hours of data with both the CGMS data and glucocard data plotted against the true glucose. The “true glucose” is denoted by the solid line and includes the modelling error of Equation (26), but not the sensor error, so that *δ* = 0 in Equation (24). The widely spread points are the simulated CGMS data which are plotted every 5 minutes using the formula in Equation (24) with  *δ* normally distributed as given in Equation (23). The circles are the simulated glucocard hourly “measurements” which put 7% random uniformly distributed noise on the “true glucose”.

The derivative method is now used in place of the integral method in Step 3 of Fig. (**3**). The same data is used, but to potentially assist the derivative method, the data is smoothed several times by a 3 point moving average. However, even with smoothing to remove most of the local noise, a significantly worse result is seen in Fig. (**18**). The mean glucose is 5.5 ± 1.1 mmol/L with only 64.86% of glucose values lying between 4.0 and 6.1 mmol/L. Thus, the derivative method is unable to take advantage of the extra CGMS data, where the integral method gives significantly better outcomes on glucose control despite the larger noise distribution for these sensors. The derivative method clearly performs better when there is very minimal modelling error and the true glucose is close to a straight line between two points, which was the case in Figs. (**11**,**13**,**15**), but does not occur with significant sensor noise and/or modelling error, both of which typically exist.

## CONCLUSIONS

This paper has reviewed the model-based therapeutics approach to glucose control that has been developed and put into regular use in the Christchurch Hospital, New Zealand ICU, and investigated the impact of two different fast parameter identification methods. The key point with these parameter identification methods is that unlike typical non-linear regression approaches, they do not require any forward numerical solutions to identify model parameters. They are also not starting point dependent, and thus provide a major advantage in the implementation of model-based “virtual” clinical trials as well as significant real-time capability. The two methods considered were a previously developed integral-based patient specific parameter identification method and a similar approach based on the derivative. At first sight it might be expected that the integral and derivative approaches would perform similarly given they are derived from the same underlying differential equation model. However, even without noise significant differences were observed for certain parameter sets and glucose control protocols, with the integral method remaining significantly more robust.

A number of tests were performed on clinically derived data from a patient in the Christchurch ICU. Very little differences were observed between the model-based glucose control using the integral method compared to the derivative method for this patient. This result is due to the fact that the insulin levels remained quite constant and high throughout, so that the insulin dynamics between measurements were minimal. The resulting glucose response was thus very close to a straight line with very little modelling error. However, when adding extra measurements from CGMS along with low frequency modelling error, the derivative method performed very poorly, and had worse results than without the CGMS. The integral method on the other hand remained robust and gave a significant improvement in glucose control.

The overall results are summarized as follows.

- The integral formulation in parameter identification is very important for robust and reliable results, particularly with respect to modelling error which is always present in clinical applications
- The derivative method is very sensitive to modelling error and only works in situations where model response is close to a straight line.
- The combination of the integral method and model-based drug control is very effective for designing and testing new protocols.

The integral method is an important research tool in the model-based therapeutics approach. For example the addition of simulated CGMS shows that a potentially significant clinical gain could be achieved with this continuous sensor. However, further investigation with real CGMS data is required to validate these results. The derivative method, went unstable and failed to realize this possible clinical gain, further emphasizing the importance of integrals in the formulation.

## Figures and Tables

**Fig. (1):**
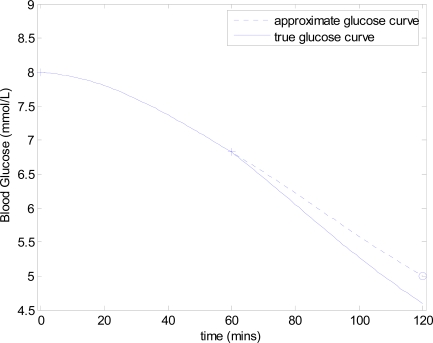
Controlling glucose to a target value of *G_target_*=5 mmol/L. The true *S_I_* in the first and second hours are defined as  *S*_*I*,1_= 0.0008  and  *S*_*I*,2_= 0.001  (L/mU min).

**Fig. (2):**
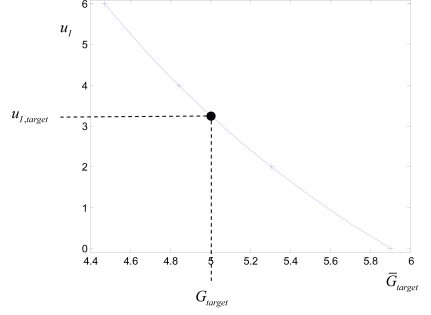
Plotting the points $\left\{\left({\bar{G}}_{\mathrm{target},1},u_{I,1}\right),...,\left({\bar{G}}_{\mathrm{target},7},u_{I,7}\right)\right\}$ from Equation (16) and fitting a cubic spline to determine *u_I,target_* in Equation (14).

**Fig. (3):**
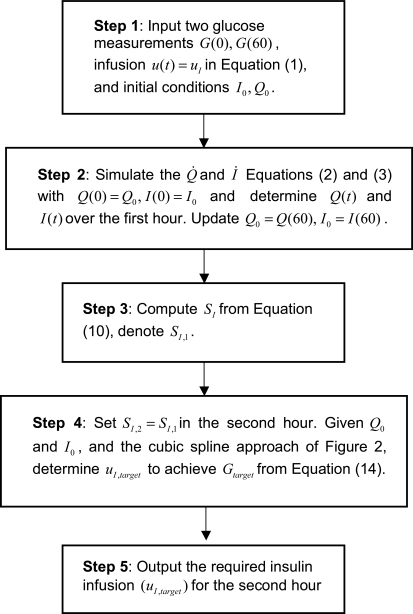
An algorithm summarizing the method of model-based glucose control which determines the required insulin infusion that brings the blood glucose to a predetermined glucose target *G_target_*. Similar approaches can be used in appropriate time frames or intervals for any drug therapy that is similarly modelled with differential equations.

**Fig. (4):**
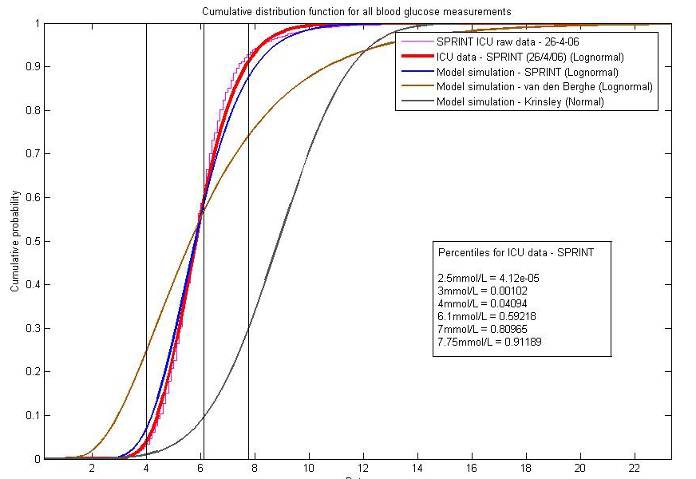
A comparison of the virtual trials approach and real clinical ICU results from SPRINT. Also shown are virtual patient simulations of two other well known protocols, Van den Berghe [46] and Krinsley [47].

**Fig. (5):**
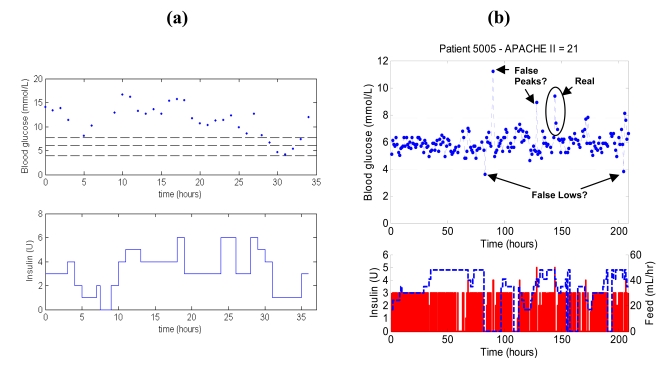
(**a**) A patient (Patient 130) on a typical sliding scale before the use of SPRINT in the Christchurch ICU. APACHE II score = 11. (**b**) A patient (Patient 5005) on SPRINT in the Christchurch ICU. APACHE II score = 21.

**Fig. (6):**
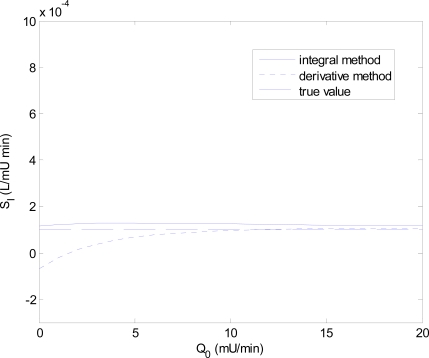
The identified insulin sensitivity  *S_I_* for the integral method of Equations (7)-(10) and derivative method of Equations (11)-(12) for the parameter set of Equation (18).

**Fig. (7):**
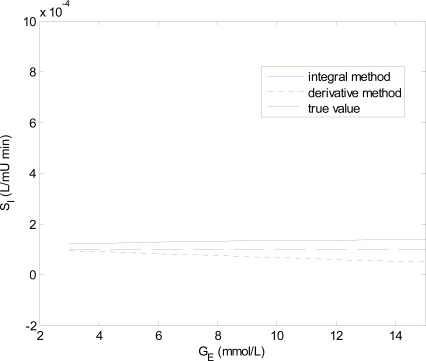
The identified insulin sensitivity *S_I_* for the integral method of Equations (7)-(10) and derivative method of Equations (11)-(12) for the parameter set of Equation (19).

**Fig. (8):**
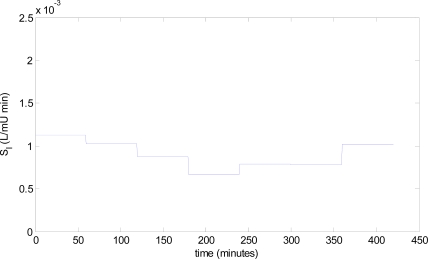
Time varying insulin sensitivity for Patient 554 from the retrospective cohort [23].

**Fig. (9):**
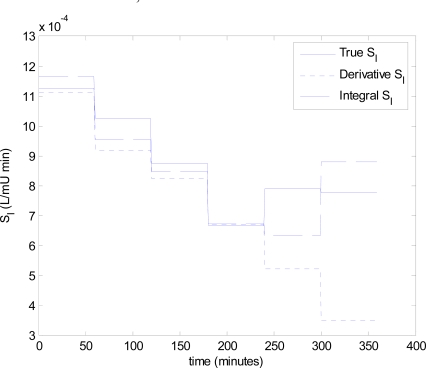
The identified insulin sensitivity values for the derivative and integral methods compared to the true insulin sensitivity for Patient 554.

**Fig. (10):**
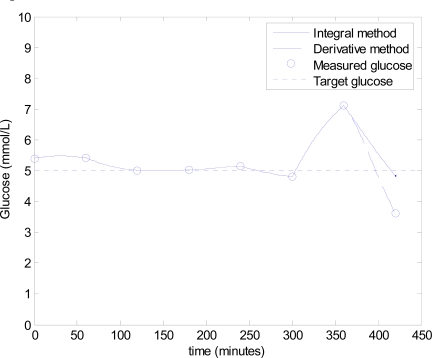
Model-based glucose control using the algorithm of Fig. (**3**), with the added constraint of minimizing the exogeneous insulin.

**Fig. (11):**
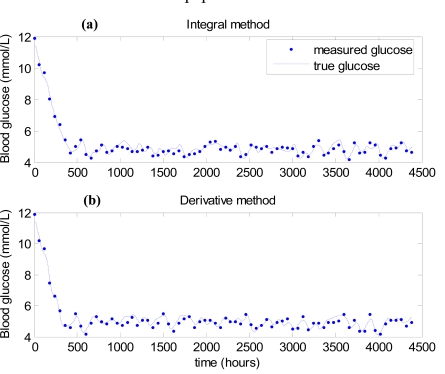
(**a**) Algorithm of Fig. (**3**), with the integral method of Equations (7)-(10)used in Step 3. (**b**) Algorithm of Fig. (**3**), with the derivative method of Equations (11)-(12) used in Step 3.

**Fig. (12):**
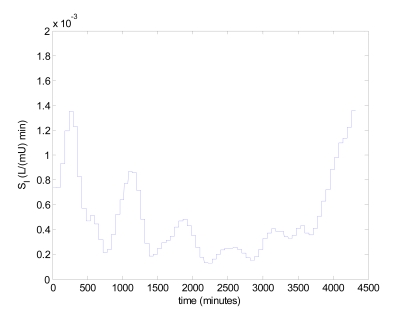
Time varying  *S_I_* profile over first 3 days for patient 519.

**Fig. (13):**
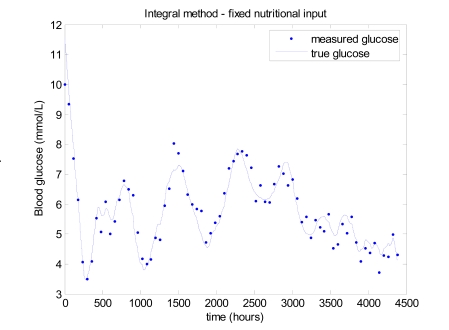
Model-based glucose control with the time varying  *S_I_*  of Fig. (**12**) and a fixed nutritional input given in Equation (22).

**Fig. (14):**
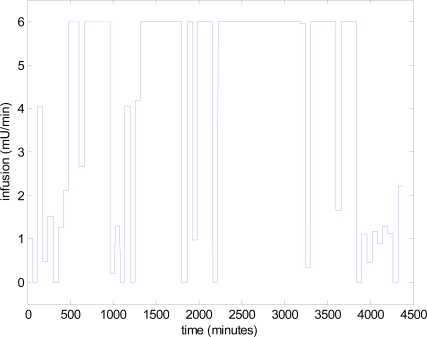
Control infusion input *u_I,target_* in Step 4 of Fig. (**3**), for the model-based glucose control of Fig. (**13**).

**Fig. (15):**
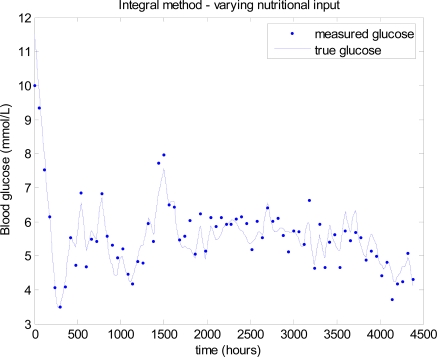
Model-based glucose control with the time varying  *S_I_* of Fig. (**12**) and a simply varying nutritional input.

**Fig. (16):**
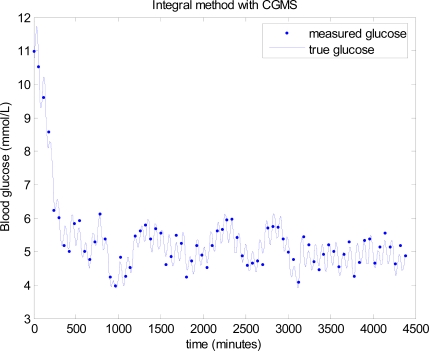
Model-based glucose control using the integral method in Step 3 of Fig. (**3**), with the combination of a CGMS sensor and glucocard.

**Fig. (17):**
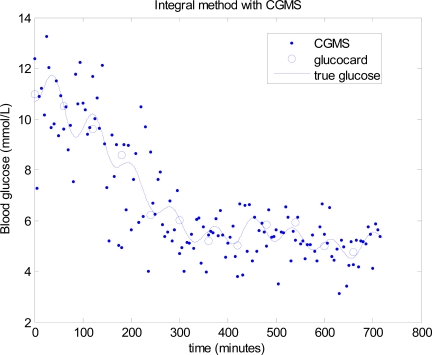
The first 12 hours (720 minutes) of patient 519, with simulated CGMS data shown as points, glucocard “measurements” shown in circles and a solid line denoting the “true glucose” which includes the modelling error of Equation (26), but not the sensor error.

**Fig. (18):**
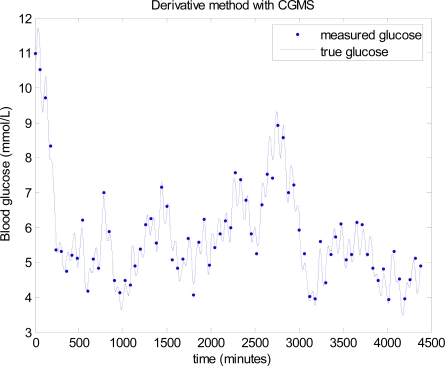
Model-based glucose control using the derivative method instead of the integral method in Step 3 of Fig. (**3**), and with a CGMS sensor and glucocard.
